# Immune Modulation by Epstein–Barr Virus Lytic Cycle: Relevance and Implication in Oncogenesis

**DOI:** 10.3390/pathogens13100876

**Published:** 2024-10-08

**Authors:** Nevena Todorović, Maria Raffaella Ambrosio, Amedeo Amedei

**Affiliations:** 1 Department of Experimental and Clinical Medicine, University of Florence, 50134 Florence, Italy; nevena.todorovic@unifi.it; 2 Clinic for Infectious and Tropical Diseases, University Clinical Centre of Serbia, 11000 Belgrade, Serbia; 3 Pathology Unit, Azienda Sanitaria Toscana Nord Ovest, 56121 Pisa, Italy; mariaraffaella.ambrosio@uslnordovest.toscana.it; 4 Network of Immunity in Infection, Malignancy and Autoimmunity (NIIMA), Universal Scientific Education and Research Network (USERN), 50139 Florence, Italy

**Keywords:** Epstein–Barr virus (EBV), lytic cycle, immunomodulation, oncogenesis, EBV-related tumors

## Abstract

EBV infects more than 90% of people globally, causing lifelong infection. The phases of the EBV life cycle encompass primary infection, latency, and subsequent reactivation or lytic phase. The primary infection usually happens without noticeable symptoms, commonly in early life stages. If it manifests after childhood, it could culminate in infectious mononucleosis. Regarding potential late consequences, EBV is associated with multiple sclerosis, rheumatoid arthritis, chronic active EBV infection, lymphomas, and carcinomas. Previous reports that the lytic phase plays a negligible or merely secondary role in the oncogenesis of EBV-related tumors are steadily losing credibility. The right mechanisms through which the lytic cycle contributes to carcinogenesis are still unclear, but it is now recognized that lytic genes are expressed to some degree in different cancer-type cells, implicating their role here. The lytic infection is a persistent aspect of virus activity, continuously stimulating the immune system. EBV shows different strategies to modulate and avoid the immune system, which is thought to be a key factor in its ability to cause cancer. So, the principal goal of our review is to explore the EBV’s lytic phase contribution to oncogenesis.

## 1. Introduction

Sixty years ago, there was a groundbreaking shift in the understanding of viruses as potential causes of tumors in humans. Antony Epstein and his team made a remarkable discovery: the Epstein–Barr virus (EBV), identified within a cell line originating from African Burkitt’s lymphoma [1]. This marked the beginning of viewing infectious agents as potential drivers of oncogenesis. Currently, a multitude of infectious agents are recognized to be linked with various cancer types [2].

Since 1964, our EBV understanding has advanced considerably, yet much remains unknown. Nevertheless, its role and mechanisms in carcinogenesis continue to be topics of ongoing research.

EBV (formally labeled as HHV-4) is a human herpesvirus, and member of the gammaherpesvirus subfamily, with a tropism for B cells and epithelial cells. It is a double-stranded DNA virus enclosed by proteins, featuring an envelope adorned with glycoproteins [3]. EBV is categorized into two types, EBV type 1 and EBV type 2, distinguished by specific genetic variations within the nuclear antigen and non-polyadenylated RNAs. While EBV type 1 is more prevalent worldwide, and demonstrates a higher efficiency in transforming B cells into immortalized lymphoblastoid cell lines compared to type 2, EBV type 2 exhibits a preference for infecting T cell lymphocytes [4]. This virus is primarily spread through saliva. Although infection cases have been reported following allograft transplantation and blood transfusion, the associated risk is actually quite low [5]. Finally, EBV can be found in secretions of the genital tract and breast milk, which are also uncommon routes of transmission [6].

EBV infects more than 90% of people globally, causing lifelong infection. It infiltrates B cells and takes residence in memory B cells. The phases of the EBV life cycle encompass primary infection, latency, and subsequent reactivation or lytic phase. During latency, the virus remains dormant for long periods, occasionally reactivating for brief episodes of lytic replication. The primary infection often happens without noticeable symptoms, commonly in early life stages. If it manifests after childhood, it could culminate in mononucleosis [7]. Regarding potential late consequences, EBV is associated with different diseases, including multiple sclerosis, rheumatoid arthritis, chronic active EBV infection, lymphomas, and carcinomas [8]. Some of the EBV-related cancers are nasopharyngeal carcinoma (NPC) and gastric carcinoma (GC); lymphomas include Hodgkin’s lymphoma (HL), Burkitt’s lymphoma (BL), diffuse large B cell lymphoma (DLBCL), the nasal type of extranodal NK/T cell lymphoma (ENKTCL), and AIDS- or transplantation-associated lymphomas. According to Wang et al., in 2020, EBV-related cases from the first six aforementioned types of cancers were estimated to contribute to 239,700–357,900 new cases and 137,900–208,700 deaths [9].

As Lieberman highlighted, some of the events that shed light on the pivotal influence of host immunity in managing viral-related malignancies were the elevated occurrence of B cell lymphomas and Kaposi’s sarcomas during the HIV/AIDS epidemic, and more notably, the EBV identification as a causative factor of X-linked lymphoproliferative disease, a genetic disorder causing immunological dysfunction [10].

In this scenario, the primary goal of our review is to finely explore and discuss the evidence of EBV’s lytic phase contribution to oncogenesis.

## 2. The Life Cycle of His Majesty, the Epstein–Barr Virus

As previously mentioned, the life cycle of EBV has two phases: latent (nonproductive) and lytic (productive) infection. Following infection, EBV delivers its linear genomic DNA of 172 kbp to the nucleus of human B cells in the oropharynx, where it forms a circular plasmid. The EBV viral particle, in addition to its double-stranded DNA, is composed of (1) a protein capsid, with the major capsid protein (VCA) being the most prominent; (2) a tegument layer that contains various viral proteins, the most relevant being BNRF1, which plays a critical role in infected cells’ transformation, along with RNA; and (3) a lipid envelope, derived from the host cell membrane, embedded with viral glycoproteins such as gp350/gp220, gB, gp42, and gp110. Each of these components plays an essential role in the EBV infection cycle, from entering the host cell to viral replication; the modulation of cells’ environment and cells’ transformation; and evading the host’s immune responses. At this stage, a phase of the EBV life cycle known as the “prelatent abortive lytic cycle” begins, during which all latent genes are coexpressed along with a limited number of lytic genes, namely BZLF1, BRLF1, BGLF4, and BFRF3 [11,12]. This phase is impermanent and no virus progeny is synthesized. Following the resolution of the primary infection, latent phase III (growth program) takes over. The expression of EBV nuclear antigens (EBNAs 1, 2, 3, A, 3B, 3C, -LP), latent membrane proteins (LMPs 1, 2A, 2B), viral noncoding RNAs (EBER1, 2), and micro-RNAs (BHRF1, BARTs) trigger the transformation of B lymphocytes into proliferating lymphoblasts [13]. After evading destruction by T cell immune response, a small subset of cells differentiates into centrocytes within the germinal centers. Those in latent phase II will enter the circulation and transition into memory B cells, serving as the primary reservoir for the EBV throughout an individual’s life [14]. At this point, the virus starts latent phase 0 (latency program). The transition of the virus into this phase is followed by a switch to a different promoter of nuclear antigen expression [15]. This shift is significant, reflecting the next epigenetic changes occurring in the viral DNA. The virus suppresses all lytic genes, while allowing the latent genes to continue functioning. The viral genome quietly replicates alongside the host cells’ DNA, using the same machinery, and no new virus particles are released. Through this strategy, the virus stays hidden from the immune system. It can persist in the latent state for the entire life of the host, intermittently producing some quantity of EBNA1 proteins to maintain replication and persistence during latent phase I, remaining undisturbed and ready to reactivate if the opportunity arises [16]. Activated by different stimuli, memory B cells differentiated into plasma cells will reactivate EBV to start the lytic cycle. During the lytic phase, which takes place in epithelial and B cells, the newly produced virion is delivered from an infected cell by exocytosis or cell lysis, ready to infect new cells or a new host. The lytic cycle is composed of three stages, immediate early (IE), early (E), and late (L) stages, represented by a significant number of viral proteins [17,18]. During the IE stage, transcription factors Zta (BZLF1 or ZEBRA) and Rta (BRLF1) start the cascade of lytic genes’ expression and viral DNA replication [19]. Early genes involved in intensive viral DNA replication include BHRF1, BALF1, BALF3, BARF1, BGLF4, BGLF5, and BMRF1. Once replication is completed, the L stage begins. Late gene products (BcLF1 and BLLF1) primarily contribute to the development of viral particles, supported by the viral pre-initiation complex (vPIC), which is composed of early genes, but specifically involves six key genes (BMLF1, BSLF2/BMLF1 complex, BFRF1, Zta, and Rta) and BGLF4. These late genes encode structural proteins like glycoprotein B (gB and gp350), major capsid protein (MCP), viral capsid antigens, and major tegument protein (BNRF1), which ultimately lead to the production of infectious viral particles and the assembly of virions. Finally, mature viral particles are created and released [20,21].

EBV plays a game of hide and seek with the human body, orchestrated through two strategies, together enabling the virus to establish a permanent base in its host. In the latent phase, the virus expresses only a minimal set of genes, just enough to keep itself alive without drawing attention, allowing it to fly under the radar of the immune system. Periodically, it activates the lytic cycle (high-profile phase) where the virus produces an eruption of new viral particles, which are primarily released in the tissue of the pharynx and salivary glands. Despite the virus’s attempts, the human immune system is no passive observer; it remains vigilant, mounting a significant response to the viral proteins produced during the lytic cycle. This response is both humoral, involving antibodies, and cell-mediated, involving immune cells directly attacking the virus. In this ongoing battle, the lytic cycle plays a crucial role. Although it exposes the virus to the immune system’s wrath, it also ensures the virus’s continued presence and ability to spread.

## 3. Hide and Seek with the Immune System

EBV shows different strategies to modulate and avoid the immune system, which is thought to be a key factor in its ability to cause cancer. In the early phase of infection, innate immune responses are initiated when PRRs (pattern recognition receptors) identify biomacromolecules with PAMPs (pathogen-associated molecular patterns) or DAMPs (damage-associated molecular patterns). Among the pivotal PRRs are Toll-like receptors (TLRs), retinoic acid-inducible gene-I (RIG-I)-like receptors, cyclic GMP-AMP synthase (cGAS), NOD-like receptors (NLRs), and C-type lectin receptors (CLRs). PRRs’ activation leads to the production of proinflammatory cytokines (IL-1, IL-6, IL-8), IFN-1, TNF-α, and chemokines through signaling pathways such as NF-kB, and the induction of interferon regulatory factors (IRF_3_ and IRF_7_). On the other hand, the EBV ability to suppress the innate immune system relies on the inhibition of inflammatory cytokine production by acting through the stimulator of interferon genes (STING) and TANK-binding kinase 1 (TBK1) signaling pathways [20,22]. Recently, Guo et al. conducted a study on the EBV-encoded envelope glycoprotein gp110. They found that gp110 is a negative regulator of host antiviral innate immunity, reporting that gp110 inhibits the promoter activity of IFN-β mediated by the RIG-I-like receptor pathway and reduces the transcription of downstream antiviral genes, thereby promoting viral proliferation. Specifically, gp110 interacts with the inhibitor of NF-κB kinase (IKKi), restraining its K63-linked polyubiquitination. This leads to a decrease in the IKKi-mediated activation of NF-κB and suppresses the phosphorylation and nuclear translocation of p65. Furthermore, gp110 interacts with β-catenin, a crucial regulator of the Wnt signaling pathway, inducing its K48-linked polyubiquitination degradation via the proteasome system, which leads to a decrease in β-catenin-mediated IFN-β production. Altogether, these findings imply that gp110 acts as an inhibitor of antiviral immunity, revealing a new mechanism of EBV immune evasion during the lytic cycle [23]. Dendritic cells activate immune cells upon detecting EBV. Recently, it has been demonstrated that the EBV abortive lytic cycle recruits monocytes and induces their differentiation toward tumor-associated macrophages (TAMs) and away from DCs. ZTA is the key regulator of monocyte chemotaxis and differentiation toward TAMs. TAMs, induced by the EBV abortive lytic cycle, in turn, promote tumor cell angiogenesis and invasion. Moreover, during lytic infection, EBV hinders the immune system to promote the maturation and release of new virions. It accomplishes this by stimulating the production of cellular cytokines such as IL-6, IL-8, IL-10, IL-13, and IL-1β [24].

Regarding adaptive immunity upon infection, B cells first generate anti-VCA IgM and IgA antibodies. Then, anti-VCA IgG antibodies reach their highest levels 2–4 weeks post-infection. CD8+ cytotoxic T (CTL) cells play a critical role by targeting EBV-infected cells through the recognition of viral peptides presented by major histocompatibility complex class I molecules (MHC-I). Meanwhile, EBV-infected cells express elevated levels of MHC-II molecules, which activate CD4+ T cells (T helper—Th). The Th cells are essential not only for aiding B cells in antibody production and antigen neutralization, but also for enhancing and sustaining the cytotoxic activity of CTL [25]. Strong evidence indicates a crucial mechanism during the lytic cycle by which EBV-infected cells evade recognition and destruction by virus-specific cytotoxic T cells: impairment of the MHC class I antigen processing pathway by reduction in the expression of MHC-I and MHC-II molecules. There are some lytic genes involved in this mechanism. In particular, BNLF2a is an E phase protein expressed in NPC, ENKTCL, and angioimmunoblastic T cell lymphoma [26]. It disrupts CTL-mediated cell lysis through HLA-A, -B, and -C alleles, downregulating levels of MHC-I by blocking TAP function (ABC transporters’ member associated with antigen processing). TAP has two binding sites for peptides and ATP, and BNLF2a inhibits both, resulting in the failure of presenting cells to CTL for their destruction. The BGLF5 gene encodes an alkaline exonuclease that suppresses cellular protein synthesis and triggers an extensive shutdown of cellular gene expression by accelerating mRNA degradation, affecting HLA-I and HLA-II molecules. This results in a significant reduction in HLA class I-restricted CD8+ T cell recognition, allowing the virus to evade the immune response. Furthermore, BGLF5 profoundly decreases both RNAs and the protein expression of TLR9 through RNA degradation. The decreased TLR9 level prevents detecting the EBV presence. In this way, the virus could impair host innate antiviral response [23]. It is still unclear if BGLF5 expression is limited to EBV-associated epithelial carcinomas. BILF1 is a protein with the ability to manipulate various intracellular signaling pathways, notably the NF-kB and cAMP-response element-binding protein (CREB) pathways. Its expression leads to the suppression of RNA-dependent protein kinase (PKR) activation, which consequently halts host protein synthesis and induces death of infected cells. Moreover, BILF1 significantly disrupts the immune response by downregulating MHC-I molecules on the surface of cells. This action accelerates their internalization and lysosomal degradation, effectively subverting the antigen presentation pathway and preventing T cell recognition of infected cells [24]. In addition, BILF1 also interacts with human chemokine receptors, forming heterodimers. Notably, it forms hetero-oligomers with the human CXCR4 receptor, which disrupts the binding of the chemokine CXCL12 to CXCR4. The BCRF1 gene encodes the IL-10 homolog, viral IL-10 (vIL-10), a cytokine with potent immunosuppressive properties. vIL-10 represents a strategic acquisition by EBV, mimicking a human cytokine to enhance viral persistence and promote cell transformation by evading the hosts’ immune responses both during the initial infection and viral reactivations. Its expression has been identified in various EBV-associated carcinomas, where it plays a crucial role in shielding EBV-infected cells from destruction by NK cells and CTLs. Specifically, it downregulates the TAP1, decreasing the transport of peptide antigens into the endoplasmic reticulum, which leads to lower surface levels of MHC class I molecules. In addition, vIL-10 diminishes the antigen-presenting capacity of monocytes by downregulating MHC class II molecules and critical adhesion and costimulatory molecules, such as ICAM-1, CD80, and CD86. The vIL-10 effects extend further, promoting the growth of infected cells while suppressing the production of key immune-stimulating cytokines like IL-2 and IFN-γ by activated T cells. This suppression not only prevents the secretion of antiviral cytokines, but also weakens the effector functions of CD4+ T cells [25]. BRLF1 undermines the host’s immune responses by interfering with the activation of inflammatory vesicles and disrupting the normal function of NK cells and T cells. It achieves this by inducing the production of proinflammatory cytokines IL-18 and IL-1β, which suppress immune cell activity. This upregulation not only blunts the activation of these key immune cells, but also contributes to their depletion, thereby weakening the overall immune response and facilitating viral persistence.

## 4. Raising a Monster: The Journey of Oncogenesis

Previous reports that the lytic phase plays a negligible or merely secondary role in the oncogenesis of EBV-related tumors are steadily losing credibility. The right mechanisms through which the lytic cycle contributes to carcinogenesis are still unclear, but it is now recognized that lytic genes are expressed in different cancer-type cells, implicating their role here. The simultaneous occurrence of the latent and abortive lytic cycle has been observed in EBV-associated cancers [27]. Studies showed that while IE and E genes were continuously detected, late genes were infrequently observed or present at low levels. These “leaky” lytic genes are expressed at modest levels during the early stages of the lytic cycle and their expression increases significantly following EBV DNA replication. This pattern of gene expression plays a big role in carcinogenesis.

Recent studies in the context of the abortive lytic cycle, which was previously considered to play a lesser role in oncogenesis, have highlighted a potential role of lytic genes in oncogenesis. It explains that, even when the full production of new viral particles is not achieved due to the viral genomes’ methylation status, certain lytic genes can still be activated. For instance, Zta selectively binds to and activates methylated promoters, leading to the expression of several early lytic genes. Its expression occurs alongside one or more early genes, but without the involvement of late structural proteins necessary for full viral replication. This incomplete lytic cycle allows for the expression of lytic genes, which in turn release paracrine signals that support tumor cell growth and facilitate immune evasion. The previously mentioned migration and differentiation of TAMs through paracrine signaling, as well as the mechanism of immune evasion by the TAP inhibition, both take place during the abortive lytic cycle. TAMs, along with other inflammatory cells, reshape the tumor microenvironment, promote angiogenesis, and play a pivotal role in promoting tumor progression. The TAP inhibition allows EBV-infected cells to remain undetected by the immune system, facilitating prolonged survival and contributing to oncogenesis without triggering a full immune response. Another crucial aspect is the role of anti-apoptotic factors, which contribute to cell survival, allowing the accumulation of mutations that eventually lead to malignant transformation. Furthermore, recent research has emphasized the epigenetic regulation that occurs during this phase. Zta has a critical role in selectively activating methylated promoters of certain lytic genes while suppressing others, which allows the virus to modulate cells’ environment. Finally, the abortive lytic cycle induces genomic instability in host cells. Repeated abortive reactivations of EBV can lead to DNA damage, chromosomal abnormalities, and other alterations. This instability may result from ROS generation, as well as from interference with the host’s DNA damage repair mechanisms. In conclusion, this phenomenon provides a potential explanation for how lytic gene expression contributes to viral tumorigenesis, without resulting in cell lysis or the release of new viral particles, which would otherwise destroy the host cell. It has far-reaching consequences in promoting tumorigenesis through a combination of paracrine signaling, immune evasion, anti-apoptotic mechanisms, epigenetic modulation, and genomic instability [24,28,29,30].

In addition to the previous mentioned two types of the virus, EBV type 1 and EBV type 2, there are variations within these strains too, which are still the subject of investigations. The main question is as follows: is there any connection between different variants and oncogenic potential? One potential explanation could be a stronger inclination towards the reactivation of the lytic cycle, which increases viral load, thus being associated with oncogenesis [31]. The right triggers of EBV reactivation in vivo during natural infections are unknown. In addition, acute infection with diverse pathogens, stress stimuli, or immunocompromised conditions may provide the suitable conditions [29,32,33]. Many chemical and biological stimulations can induce reactivation at the cell culture level [34]. Thus, a potential mechanism through which the reproductive cycle contributes to the carcinogenesis is by enhancing the virus’s ability to spread from one cell to another to increase the total number of latently infected cells [20,26]. Speculations regarding this hypothesis emerged soon after the discovery of EBV as the first human tumor virus. The pioneering study, launched in Uganda in 1972, sought to explore the link between Burkitt’s lymphoma and EBV. Over a span of seven years, this study revealed a promising correlation, showcasing serological evidence of elevated antibody titers to EBV’s VCA prior to the onset of Burkitt’s lymphoma in the bloodstream of numerous children [20].

To date, several potential mechanisms underlying the development and progression of EBV-induced carcinomas have been identified. No single mechanism has been confirmed to dominate. It is most likely a synergistic interplay of the indirect or direct promotion of cell proliferation, genomic instability, anti-apoptotic and angiogenic signals, angiogenesis, and inflammation that impact the tumor microenvironment and cytokines’ production. Finally, the predominant role of the EBV lytic cycle is likely to provide the necessary signals required for this process (Table 1).

Genomic instability in cells is one of the fundamental characteristics that facilitates the onset of cancers and has been recognized in EBV-induced carcinomas since the early days following the discovery of this virus [35,36,37]. It is characterized by the occurrence of microsatellite and chromosomal instability, and alterations of different mechanisms involved in DNA damage repair and chromosomal segregation. Studies have demonstrated that EBV replication induces genetic instability in host cells, promoting a malignant phenotype, even after losing EBV infection. Gargouri et al. demonstrated DNA damage 48 h after the EBV lytic cycle induction (which presents the peak of the lytic cycle). They observed DNA fragmentation caused by reactive oxygen species (ROS) production in two lymphoblastoid cell lines [38]. Fang et al. conducted a study using an in vitro system that compared EBV+ and EBV− nasopharyngeal carcinoma cells. They demonstrated that repeated EBV reactivations, induced by 12-O-tetradecanoylphorbol-13-acetate and sodium n-butyrate, significantly affect genome stability, cause chromosomal aberrations, and enhance malignant characteristics in nasopharyngeal carcinoma cells [39]. Numerous studies have been carried out on this topic [40,41,42]; some of them are reviewed by Rosemarie et al. [36]. Regarding the EBV ability to deregulate the DNA damage response (DDR), which is signaling pathway-activated also during the lytic reactivation of EBV, Hau et al. provided a review explaining the mechanisms and the relationship between EBV-encoded viral proteins and the DDR transducers, including ATM (ataxia telangiectasia mutated), ATR (ATM and Rad3-related), and DNA-PK (DNA-dependent protein kinase) [43]. It has been documented that some of the lytic genes foster genomic instability [15]. BGLF5 triggers DNA double-strand breaks, leading to micronuclei formation, genomic instability, and increased chromosomal aberrations by damaging DNA and inhibiting repair mechanisms. BALF3 also induces DNA damage through its nuclease activity, enhancing cell migration, invasion, spheroid formation, and tumor growth. BNRF1 causes centrosome amplification, aneuploidy, and the production of a truncated PARP1, which contributes chromosomal instability. In addition, BNRF1 disrupts cellular antiviral defenses, interacting with DAXX to disassemble the DAXX-ATRX complex and promote viral latency, B cell proliferation, and EBV-induced immortalization [44]. As far as ZEBRA is concerned, it induces oxidative stress, which increases genomic instability. The IE gene BRLF1 also induces chromosomal misaggregation and genomic instability by activating the Erk signaling pathway.

The discovery of the first viral microRNAs (miRNAs) in 2004 within the EBV stemmed from a Burkitt’s lymphoma cell line [45]. These viral miRNAs integrate into the RNA-induced silencing complex, which targets the 3′ untranslated regions of both host and viral mRNAs [46]. This interaction suppresses target gene expression through translational repression or mRNA degradation, aiding in the maintenance of latent EBV infection, evasion of the host immune system, and promotion of tumorigenic growth in infected cells. EBV miRNAs are pivotal in carcinogenesis, influencing key biological processes that drive cell transformation and tumor progression. EBV miRNAs are categorized into two groups: BHRF1 and BART miRNAs. BHRF1 miRNAs are observed in de novo infection, latency III, and after reactivation, while BART miRNAs are present throughout latency 0, I, II, and III and highly prevalent in gastric carcinoma and NPC. The transition from latent to lytic infection is controlled by BHRF1-2-5p miRNA and BART2-5p miRNA, which target key molecules in B cell receptor (BCR)-mediated signaling. These molecules encompass GRB2 (Growth Factor Receptor-Bound Protein 2), MALT1 (Paracaspase MALT1), RAC1 (Rac Family Small GTPase), and SOS1 (Rac Guanine Nucleotide Exchange Factor 1). During lytic infection, EBV miRNAs (1) suppress apoptosis, (2) promote the proliferation of infected cells, (3) alter the host immune system, and (4) create an inflammatory environment that supports vascular remodeling and immune evasion [47,48]. EBV cleverly modulates host miRNAs and co-opts signaling networks to transform infected cells. Over 60 host miRNAs are altered, including miR-155, miR-146a, miR-21, miR-17/92, and miR-34a. miR-34a is upregulated due to the DNA damage response triggered early in infection. miR-155 is upregulated through the action of CD40-mimic LMP1 and EBNA2. In addition, LMP1 and EBNA2 influence miR-21 and miR-146a, induced by EBV reactivation. LMP1 and EBNA3A/C boost the levels of miR-221/222 [49]. Given that miR-21-5p is one of the most commonly deregulated microRNAs in cancers, Koleśnik et al. performed a study to investigate its role in EBV-associated oropharyngeal squamous cell carcinoma. Their findings revealed significantly higher values of miR-21-5p, as well as an increased grading and TN stage, in the EBV (+) group. In addition, they observed positive correlations between miR-21-5p and IL-10, MMP (matrix metalloproteinase)-3, and MMP-9, and a negative correlation between miR-21-5p and TLR9 [50].

During early lytic replication, the microenvironment is shaped by the migration of monocytes, which then differentiate into immune-suppressive tumor-associated macrophages through the action of CCL5 or viral macrophage inflammatory proteins. EBV diminishes the immune response mediated by CD8^+^ T cells by inhibiting the CXCL11-driven recruitment of these cells and decreasing MHC class I-restricted antigen presentation via its miRNAs, expressed during both latency and lytic infection. Gp42 interacts with HLA class II/peptide complexes to prevent the activation of CD4^+^ T cells that viral IL-10 homolog BCRF1 inhibits and modulates their function. In addition, EBV-infected cells interact with dendritic cells (DCs) in two distinct ways: they can suppress DC function to evade the host immunity using galectin-1 (protein within exosomes); or they can enhance DC activation through EBERs in exosomes, leading to T cell activation and a systemic production of proinflammatory cytokines. Moreover, in Hodgkin’s lymphoma, tumor cells and inflammatory infiltrates produce TGF-β, which likely plays a role in inducing regulatory T cells (Tregs). Besides the aforementioned immune cells, other microenvironment factors have a big impact. Some of them have a proinflammatory effect, such as the inhibition of the IFN-γ signaling pathway by BLZF1 encoded by EBV-infected cells. In addition, the CXC chemokine family member IP-10, which plays a role in chemotaxis, apoptosis induction, and cell proliferation, can be mediated by LMP1, or indirectly by IFN-γ. Zta incites IL-8 expression and secretion, and elevates levels of TGF-β mRNA and active TGF-β. dUTPase (BLLF3) urges the expression of proinflammatory cytokines like TNF-a, IL-1β, IL-6, and IL-8. Conversely, others have an immunosuppressive effect, such as IL-10 produced by various immune cells, and tumor growth factors elevated in EBV-positive tumors such as IL-4 and IL-13 [51,52]. Lysis of EBV+ cells can create a tumorigenic microenvironment by triggering cell signaling molecules, growth factors, and cytokines that drive genomic instability, angiogenesis, and cell proliferation. In addition, the increase in the number of latently infected cells also contributes to the tumors’ development by affecting neighboring cells [53].

Apoptosis and autophagy constitute functionally distinct mechanisms for the destruction of cytoplasmic pathogens within a cell. In case of EBV, two anti-apoptotic proteins, BHRF1 and BALF1, prevent cell death early in infection, aiding in the transformation and survival of infected cells, which contributes to cancer development. BHRF1 targets mitochondria, stabilizing them to prevent apoptosis and promoting tumor growth through mitochondrial autophagy (“mitophagy”). In detail, BHRF1 interacts with Bim and induces mitochondrial membrane permeabilization transition, leading to increased ROS production. Furthermore, this anti-apoptotic protein interferes with the immune response by promoting mitophagy, inhibiting IFN production, and stimulating viral replication. BALF1 influences autophagy, likely to support viral replication while promoting cell migration, invasion, and metastasis [54].

It is known that EBV promotes angiogenesis, degrades the extracellular matrix (ECM), and modulates epithelial–mesenchymal transition (EMT), processes that facilitate tumor growth, invasion, and metastasis. Specifically, BILF1 promotes the secretion of vascular endothelial growth factor (VEGF), which is a stimulant of blood vessel formation. BZLF1 and BRLF1 together have key roles in upregulating matrix metalloproteinases (MMPs) like MMP1, MMP3, and MMP9. Moreover, BZLF1 upregulates TIMP-1 in B cells, an inhibitor of MMPs, which is anti-angiogenic but also supports tumor cell survival by preventing apoptosis. Thus, EBVs’ lytic phase influences angiogenesis and cell invasion, primary through BZLF1 and BRLF1, with possible involvement of other lytic factors [55]. In a study conducted by Lui et al. on EBV-associated gastric carcinoma (EBVaGC), a lower expression of endothelin-1 (ET-1) was found in EBVaGC patients compared to those with EBV-negative gastric carcinoma. Reduced ET-1 expression was linked to fewer instances of lymph node metastasis. Additionally, the study explored the ET-1/endothelin receptor type A (ETAR) axis, finding that inhibiting the ERK1/2 pathway downregulated ET-1 and FOX01, suggesting ET-1’s role in promoting cell proliferation, migration, and anti-apoptosis in gastric cancer through the ET-1/ETAR axis [56].

“Hit and run” is one intriguing theory that suggests that the EBV promotes cancer development through several steps: (1) The virus initially infects cells and uses viral proteins to modulate cellular pathways involved in cell proliferation, apoptosis, and immune evasion, ultimately leading to the immortalization of the infected cells. (2) The viral DNA is then lost during cell division or due to other factors. (3) Despite the loss of viral DNA, the transformed cells persist. These transformations include alterations in gene expression, epigenetic modifications, and the dysregulation of signaling pathways, which together contribute to the cells’ transformed phenotype. (4) The cellular changes may have already primed the cells for malignant transformation. Without the viral DNA, there is no need for the virus to maintain a latent infection, allowing the transformed cells to proliferate autonomously [18,57,58,59,60,61]. Srinivas et al. demonstrated that the total and spontaneous loss of the EBV episome in a BL cell line did not affect its tumorigenic characteristics [59]. Additionally, the loss of the tumorigenic phenotype did not occur in BL cells after cloning in soft agar with the loss of EBNA1 expression [58]. Furthermore, the same phenotype was retained in gastric and nasopharyngeal carcinoma cells grown in tissue culture [62]. Finally, the maintenance of tumorigenic characteristics was also observed in two studies where EBV DNA, EBER, LMP-1, and EBNA proteins were detected in early Hodgkin’s lymphoma. Repeated analyses revealed that these proteins were missing in the relapsed tumor [60,63]. The essence of the “hit and run” theory is that although EBV is crucial in the initial transformation of cells, the subsequent loss of viral DNA does not diminish its contribution to oncogenesis. In practical research, this theory highlights that to detect this virus and EBV-related products, in addition to routine methods like immunohistochemistry and EBV-encoded RNA (EBER) in situ hybridization (ISH), which have low specificity and reliability, more sensitive tools such as EBV-microRNA detection, EBV load measurement, and EBV mRNA detection should be used [64,65,66]. On the other hand, there are studies with no evidence to support this theory, such as the study conducted by Gallagher et al. [67].

## 5. Conclusions

Through the elegant choreography of its productive and nonproductive phase, EBV assumes the role of a skillful conductor in its interaction with the host organism. It adeptly evades detection by the immune system and unleashes its full potential when the conditions arise. It transitions from a seemingly innocuous virus in the realm of “asymptomatic infections” to inducing relatively benign ailments such as infectious mononucleosis and oral diseases, all the way to multiple sclerosis, and ultimately culminating in the direst of outcomes—EBV-associated malignancies. Accumulating evidence suggests that EBV-encoded lytic genes may drive cancer development through various processes. These include generating infectious particles during the primary infection or lytic reactivation to spread the virus and transform the cells, and controlling oncogenic pathways via lytic proteins and miRNAs.

**Figure 1:**
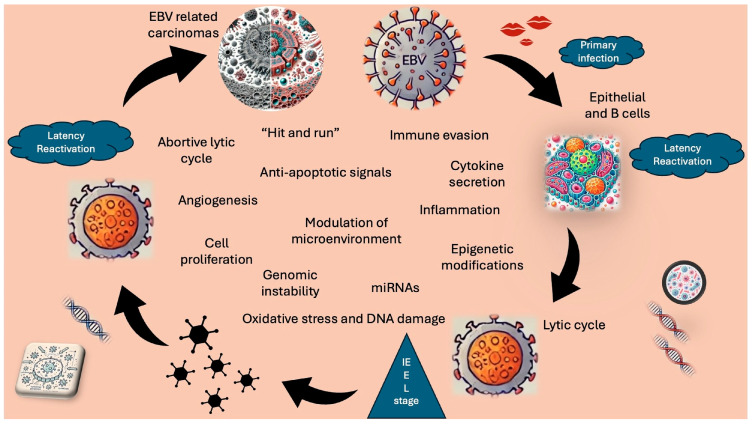
Presentation of EBV-related oncogenesis with correlated mechanisms, induced by its lytic cycle.

Some effects influence only infected cells, while others affect neighboring cells or alter the tumor microenvironment. On the other hand, had the virus not been such a masterful immune system modulator, the subsequent consequences would never have arisen. Each of the factors and mechanisms discussed in this review has a contribution in EBV-associated carcinogenesis, alongside numerous others that were not mentioned (Figure 1). For decades, the cascade triggered by this virus has shown us that it is insufficient to focus on only one aspect. Given the significant number of deaths mentioned in the introduction (137,900–208,700), it is essential to invest additional effort in finely understanding the mechanisms through which EBV contributes to the development of lymphomas and carcinomas. The relatively fresh area of research focused on the lytic cycle presents opportunities for developing novel therapeutic methods. The immune system, which plays a documented and critical role in these processes, is a target for existing treatments. Current strategies for immunotherapy are vaccination, adoptive cell therapy, and immune checkpoint blockades. Immunotherapy possesses great potential to overcome the limitations of traditional approaches but to achieve success, we must continue to delve deeper.

## Figures and Tables

**Table 1:** EBV lytic proteins and tumorigenic functions.

Cellular Homologs	Lytic Function	Mechanisms of Oncogenesis	Oncogenic Function	EBV Lytic Gene
c-Fos, c-Jun	IEA, transactivator	Enhances the release of proangiogenic factors, cytokines (VEGF, IL-6, IL-8, IL-10, IL-13), toxoid protein		ZEBRA (Zta, BZLF1)
			Angiogenesis, B cell growth, immunomodulation	
Bcl-2	EA, delay cell death	Inhibition of apoptosis	Cell survival	BHRF1 and BALF1
G protein-coupled receptor	EA, immunoevasion	MHC class I downregulation		BILF1
	EA, nucleocapsid trafficking	Engages with centromeres, leading to centrosome overduplication	Genome instability	BNRF1
Terminase	EA, DNA synthesis, incorporation into virions	DNA damage	Genome instability	BALF3
	EA, DNA replication, nuclear import	DNA damage	Genome instability	BGLF4
C-fms receptor	EA, immunomodulator	Regulates the expression of genes involved in apoptotic signaling	Cell survival	BARF1
	Host cell shutoff, TLR-9 downregulation	DNA damage	Genome instability	BGLF5
Il-10	Immunoevasion	Downregulates interferon gamma	Cell survival	BcRF1

EA, early antigen; IEA, immediate early antigen.

## Data Availability

No new data were created or analyzed in this study.
